# Clinical, angiographic, and electrocardiographic predictors of infarct size and microvascular obstruction sssessed by MRI

**DOI:** 10.1186/1532-429X-11-S1-O28

**Published:** 2009-01-28

**Authors:** Holger Thiele, Josef Friedenberger, Kathrin Schindler, Ingo Eitel, Eigk Grebe, Matthias Gutberlet, Gerhard Schuler

**Affiliations:** University of Leipzig – Heart Center, Leipzig, Germany

**Keywords:** Percutaneous Coronary Intervention, Infarct Size, Left Ventricular Mass, Primary Percutaneous Coronary Intervention, STEMI Patient

## Introduction

Infarct size (IS) and presence of microvascular obstruction (MO) assessed by delayed enhancement MRI are associated with major adverse events in ST-elevation myocardial infarction (STEMI). The time-to-reperfusion, electrocardiographic and angiographic parameters are also of prognostic relevance in STEMI patients. Predictors of IS and MO occurrence have not been assessed so far.

## Purpose

To assess predictors of IS and MO in a large consecutive series of patients with STEMI.

## Methods

This study analyzed 358 consecutive STEMI patients reperfused by primary percutaneous coronary intervention within 12 hours after symptom onset. IS and MO were assessed by delayed enhancement MRI as percentage of left ventricular mass (%LV) 3.1 ± 4.1 days after the index event. Reperfusion times, 90 min ST-segment resolution, TIMI-flow grades pre and post PCI, TIMI risk score and multiple clinical parameters such as cardiovascular risk factors, Killip-class, and infarct location were assessed.

## Results

In patients with pre PCI TIMI flow 0–1 IS was significantly higher with 24 ± 14% versus 15 ± 14% in TIMI-flow 2–3 (p < 0.001). Similarly, the extent of MO occurrence was affected by the pre PCI TIMI flow. The post PCI TIMI flow had no significant effect on final IS and MO occurrence. In patients with TIMI flow <3 IS was 28 ± 11% versus 20 ± 12% in TIMI-flow = 3 (p = 0.05). The ST-segment resolution correlated inversely with final IS and presence of MO (IS r = -0.34, p = 0.003; MO r = -0.31, p = 0.004). Anterior MI IS was 25 ± 16% (MO 7.8 ± 9.8%) versus 17 ± 12% (MO 3.8 ± 4.7%) in inferior MI (p = 0.002 IS; p = 0.003 MO). According to sixtiles of time-to-reperfusion, there was no interaction between time-to-reperfusion and IS and extent of MO even when restricted to patients with pre PCI TIMI flow 0–1. In a multivariable model the strongest predictors of IS and MO were pre-PCI TIMI-flow, infarct location, Killip class, and 90 minute ST-segment resolution (p < 0.05 for all).

## Conclusion

The pre-PCI TIMI flow, infarct location, Killip class and ST-segment resolution are the strongest predictors of IS and extent of MO. This may explain why these clinical, angiographic and electrocardiographic measures are associated with survival. In contrast to other studies the time-to-reperfusion did not affect IS and MO, which might be a selection bias, as patients with larger infarctions will be treated earlier.

